# Paradigm shift in future biophotonics for imaging and therapy: Miniature living lasers to cellular scale optoelectronics

**DOI:** 10.7150/thno.75905

**Published:** 2022-10-17

**Authors:** Paromita Sarbadhikary, Blassan P. George, Heidi Abrahamse

**Affiliations:** Laser Research Centre, Faculty of Health Sciences, University of Johannesburg, P.O. Box 17011, Johannesburg 2028, South Africa.

**Keywords:** Biological lasers, Bioimaging, Biophotonics, Laser, Nanomaterials, Optoelectronic, Optogenetics

## Abstract

Advancements in light technology, devices and its applications have tremendously changed the facets of biomedical science and engineering to provide powerful diagnostic and therapeutic capabilities ranging from basic research to clinics. Recent novel innovations and concepts in the field of material science, biomedical optics, processing technology and nanotechnology have enabled increasingly sophisticated technologies such as cellular scale, wireless, remotely controlled micro device for *in vivo* integrations. This review deals with such futuristic applications of biophotonics like miniature living lasers, wireless remotely controlled implantable and cellular optoelectronics for novel imaging, diagnostic and therapeutic applications. We begin with an overview of the competency and progress in biophotonics as one of the most active frontiers in advanced analytical, diagnostic and therapeutic modalities. This is further followed by comprehensive discussion on recent advances, importance and applications, towards miniaturization size of laser to integrate into live cells as biological lasers, and wearable and implantable optoelectronic devices. Such applications form a novel biocompatible platform for intracellular sensing, cytometry and imaging devices. Further, the opportunities and possible challenges for future research directions to transform this basic research to clinical applications are also discussed.

## Introduction

**Figure 1:**
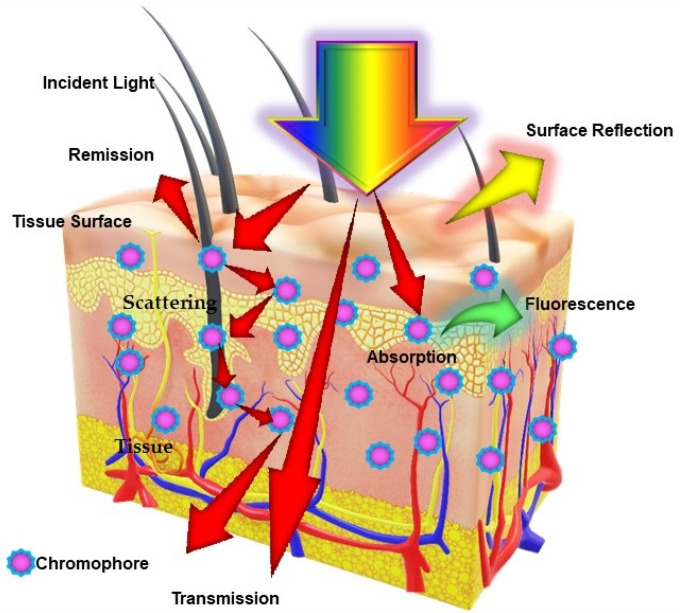
Schematic representation of different types of light interaction with a biological tissue: Excitation of biological tissue syrface with a incident light beam resulting into several events which includes absorption, fluorescence, reflectance and scattering, depending on the optical properties of tissue and content of biological chromophores.

**Figure 2:**
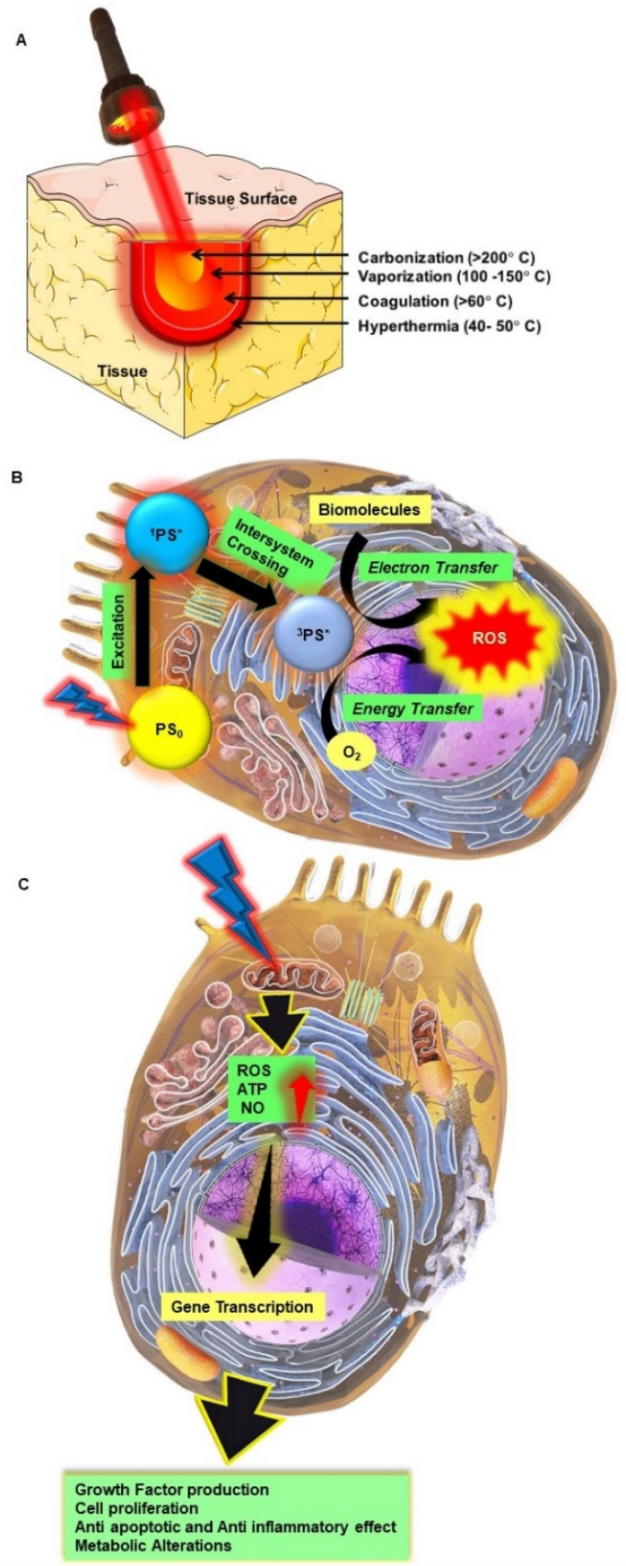
Direct and indirect effects of high power and lower light radiation on biological tissues and cells. A Photothermal therapy; B Photodynamic therapy; C Photobiostimulation.

The term photonics coined by the French physicist Pierre Aigrain in 1967, is a branch of science and technology that deals with all methodologies and technologies of light and its interaction with any matter 1. While biophotonics, a multidisciplinary scientific field combines light-based technology particularly with biological materials and biomedical applications. Over the past 30 years, we have seen great progress in the field of biophotonics and has emerged as one of the most active frontiers in advanced analytical, diagnostic and therapeutic modalities of the 21^st^ century. Biophotonics uses innovative and transformative light-based technologies for understanding and manipulating biological systems at the molecular, subcellular, cellular, tissue, and organ levels in a non-invasive manner, which exclusively depends on the interaction of light with biomolecules and tissues at nano, micro and macro scales as represented in Figure 1 2. Biophotonics based optical techniques used for biomedical imaging and sensing includes diffuse optical tomography, multiphoton microscopy, multimodal macroscale imaging, near-infrared spectroscopy, optical coherence tomography, optical tweezers and vibrational imaging for non-invasive diagnosis and intraoperative imaging 3,4. Moreover, over the years, biophotonic imaging has emerged as a potential clinical intraoperative tool for the assessment of carcinoma both at the microscopic and macroscopic scale. As an example, near-infrared (NIR) light based optical biopsy can provide micron-scale resolution in imaging which allows a less precise delineation of tumor margins and lymph nodes 5. Likewise, NIR based optical mammography allows gaining spectroscopic information and three-dimensional (3D) images of whole breast tissues 6. As represented in Figure 2, therapeutic biophotonics involves applications of light either for (a) direct interaction with cells or tissues via photothermal therapy and photo-ablation (Figure 2A) such as in laser-tissue weld and laser surgery and/or (b) indirect interaction via light-mediated photochemical reactions or signaling such as in photodynamic therapy (Figure 2B) and photobiostimulation (Figure 2C) (Low-level laser therapy) 7,8.

As per market analysis, the global biophotonics market size is expected to reach $63.1 billion by 2022 from $31.6 billion in 2015 9. Application of biophotonics available in the biomedical market includes surface imaging, inside imaging, see-through imaging, microscopy, biosensors, light therapy, spectromolecular and analytical sensing. Among all these applications, see-through imaging comprises the largest market segment in biophotonics and has emerged as an effective tool for *in vitro* and *in vivo* imaging. While on the other hand due to widespread applications and advances in endoscopic technique as minimally invasive procedures for treatment and diagnosis has led to surface imaging as the fastest growing segment 10,11. The breakthrough discovery that has tremendously changed the paradigm of biophotonics involves Laser, Laser technology and their applications in biomedical applications. Lasers have been shown to play an integral role in every field of biophotonics ranging from microscopy, cytometry, spectroscopy, and medical imaging to therapy. The global Medical Laser Market size is estimated to reach $10.57 billion by 2027 from $4.37 billion in 2019. Where the surgical lasers segment accounts for the second largest medical laser market share followed by dental lasers 12,13.

Biophotonics is no longer confined to optical properties of light and simple engineering technology. The introduction of nanotechnology in biophotonics has greatly expanded various novel scope and unique capabilities in diagnostics to therapeutics. The use of nanostructured materials such as robust contrast agents, fluorescent probes, sensing substrates and quantum dots have already revolutionized the field of biophotonics 14,15. Besides these nanomaterials, novel concepts and developments in nanophotonics is an emerging field that have great potential to enable advancements in medical lasers and optoelectronic-based medical devices down to the cellular scale. Nanophotonics which is basically photonics at the nanoscale is the science and engineering of light-matter interactions that take place on wavelength and subwavelength scales where the physical, chemical, or structural nature of natural or artificial nanostructure matter controls the interactions. The nanophotonic structures and/or devices offers the advantages of low power dissipating densely integrated information systems, tremendous reductions in energies for device operation, enhanced spatial resolution for imaging and patterning, and improved sensitivity and specificity of detection, high throughput, ease-of-use and miniaturization. 16. These properties allow probing diseases at the nanoscale, and characterize the minute responses of individual cells and biomolecules in the micro, nano, and even picoscale range. Further, nano bio-structures consisting of metallic or semiconducting nanomaterials display certain exciting functions such as a massive surface-to-volume ratio, unique optical, chemical, and electronic properties easy engineering and functionalization, high level of structural diversity and tunability, all these properties thus modify their physico-chemical properties due to their nanoscale organization, thus exhibits the potential for applications in nano-biophotonics. Merging photonics and nano-biosciences provides a powerful platform to combine photonics as a tool to investigate nano-biosciences and related novel materials as well as exploring the linear and nonlinear optical properties of these materials to widen the frontier of the most recent photonics achievements 17. Moreover, compared to current conventional macroscale technology for imaging and therapy, nanophotonic applications offers several advantages of (i) need of minute amount of sample and testing agents; (ii) fast analysis and processing time; (iii) improved accuracy with highly sensitive; (iv) increased portability, allowing point-of-care diagnostic in the form of micro and nano-fabricated tools; and (vi) low cost 18. Use of nanophotonics have promoted developments and advancements of several different technologies for molecular diagnostics and therapeutics like Localized Surface Plasmon Resonance, Raman-Spectroscopy, NIR based Fluorescence, Optical Coherence Tomography, Photoacoustic Imaging, Two-Photon Luminescence, Plasmonic Photothermal Therapy and Photodynamic Therapy for therapy as well as for combined diagnostics/therapeutics (theranostics) 19-21.

The field of nano-biophotonics is much broader hence this review tries to create a constructive overview of some of the major advances in certain important selected topics in the field of miniature living lasers, wireless remotely controlled implantable and cellular optoelectronics. Some reviews published in the field of biolasers and integratable optoelectronic microprobes focusing more on their basic concepts, designing strategies, potential multifunctional materials and their potential biological and biomedical applications in bio and/or chemical sensing 22-32. However, this review majorly focused on providing a comprehensive discussion on more recent advances and state-of-the-art techniques for miniaturization of the biological lasers and optoelectronics down to the *in vivo* cellular and implantable levels emphasizing their potential applications as micro and nano-fabricated tools with respect to present and future diagnostics and therapeutics.

## A trip through the Laser Technology

The LASER (Light Amplification by Stimulated Emission of Radiation) technology celebrated its 60^th^ anniversary in 2020. The conceptual foundation of Laser technology dates back to 1916, when for the first time Albert Einstein proposed the concept of “Stimulated Emission” stating that the photons could stimulate the emission of identical photons from excited atoms. His concept of “Stimulated Emission” became the foundational principle of all laser operations 33. The term LASER was coined by Charles Townes in 1951. But it took almost 40 years to build first working prototype of a laser. The first working laser was developed by Theodore Maiman in 1960 at Hughes Research Lab in California which was based on synthetic ruby as the lasing medium and emitting a deep red beam of light of 694.3 nm wavelength. Following this Theodore Maiman and Arthur Schawlow, stated that 'the laser is a solution in search of a problem'. 1960s proved to be the miraculous decade in the history of lasers as various lasers based on solid, gaseous, semi-conductor and liquid lasing media were invented. Since then, the advancement and newer inventions in laser technology has become one of the most rapidly growing fields in fundamental and applied research to date and will keep on doing so in the future as well. In comparison to a conventional light source, the distinctive qualities of laser to generate an intense, very narrow beam of light of a single wavelength, have been harnessed for various applications in scientific research, telecommunications, technology, medicine, materials science and many more 33-36.

**Figure 3:**
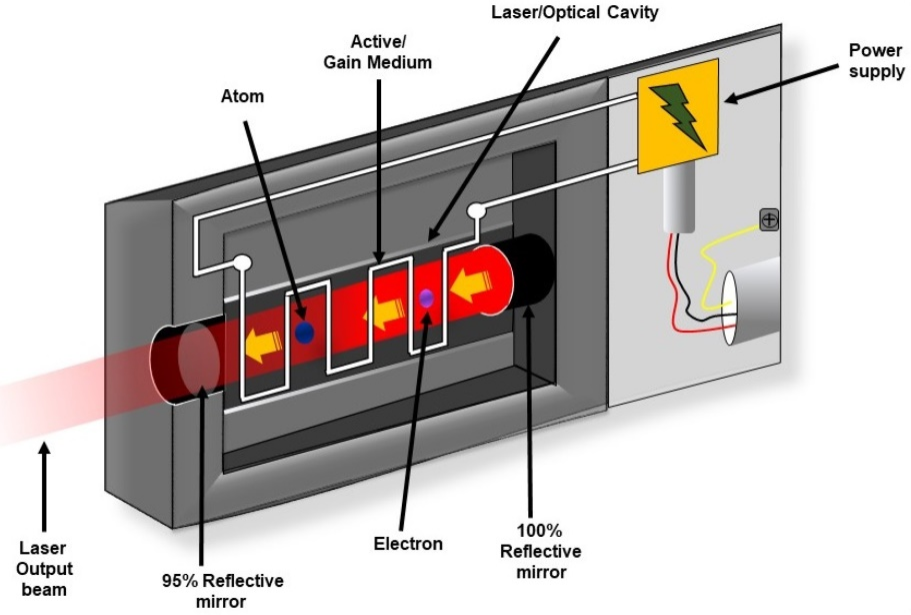
Schematic representation of components of laser systems. The critical components of a laser include a gain medium, a pump source, and a resonator.

As shown in Figure 3, every laser consists of three basic components: (a) Lasing material or active medium, (b) external energy source, and (c) optical resonator. The active medium is a suitable material where a population of excited state can be made higher than the population of ground state by pumping. The phenomenon is known as population inversion. Conventional laser amplification usually uses gases (He, Ne, CO_2_, etc), organic dyes, semiconductors and solid materials (YAG, sapphire (ruby) etc. as lasing materials 37. The excited state populations then relax to ground state via emission of electromagnetic wave or photon. The external energy source or pumping is a means to excite the active medium heavily to achieve population inversion. This means can be by electrical discharge, flash lamp or another high intensity laser. The optical resonator is a mirror-based cavity within which the active medium is placed. The arrangement filters a particular set of electromagnetic wave or photons to amplify their number by multiple reflection and repetitive passing through the active medium 37-40.

### Lasers in biomedical applications

Along with the invention of Lasers, application of Lasers in medicine shares a history of 60 years. Soon after the invention of first working prototype of ruby laser in 1960, Dr. Leon Goldman reported the first ever application of lasers in medical field in 1962. For which, Dr. Leon Goldman, is regarded as the “father” of lasers in medicine and surgery. He used lasers for dermatology, to treat a variety of pigmented lesions (melanoma) and showed selective destruction of skin pigmented structures by ruby lasers. Following which in 1963, McGuff used ruby laser in cardiovascular surgery for ablation of atherosclerotic plaques. With encounter of difficulties of ruby laser for controlling the power output, like light delivery rate and poor absorption by some types of tissues, necessitated the discovery of new laser types. Later, in 1968, F. L'Esperance, E. Gordon, and E. Labuda successfully used an argon ion laser for the treatment of diabetic retinopathy. The surgical uses of various types of lasers were investigated extensively from 1967 to 1970 by many great pioneers T. Polanyi, G. Jako, V. C. Wright and I. Kaplan in otolaryngology, gynecological and latter for general surgeries. After more advancement in laser technology and longtime of investigations, first clinical applications of lasers were performed by Choy and Ginsburg in 1983 for angioplasty in coronary and peripheral vessel 41,42. All these encouraging results brought a new branch of science dealing with the applications of 'laser medicine'. With the successful discovery of new laser types along with development of laser physics and new ways of using lasers such as the generation of radiation with various energy levels, new wavelengths, high power and small beam divergence, influenced a lot for newer areas in medical treatments till date 43,44. Table 1 represents list of commonly used lasers for medical applications. Different wavelengths of lasers used for several medical applications are listed in Table 2.

**Figure 4:**
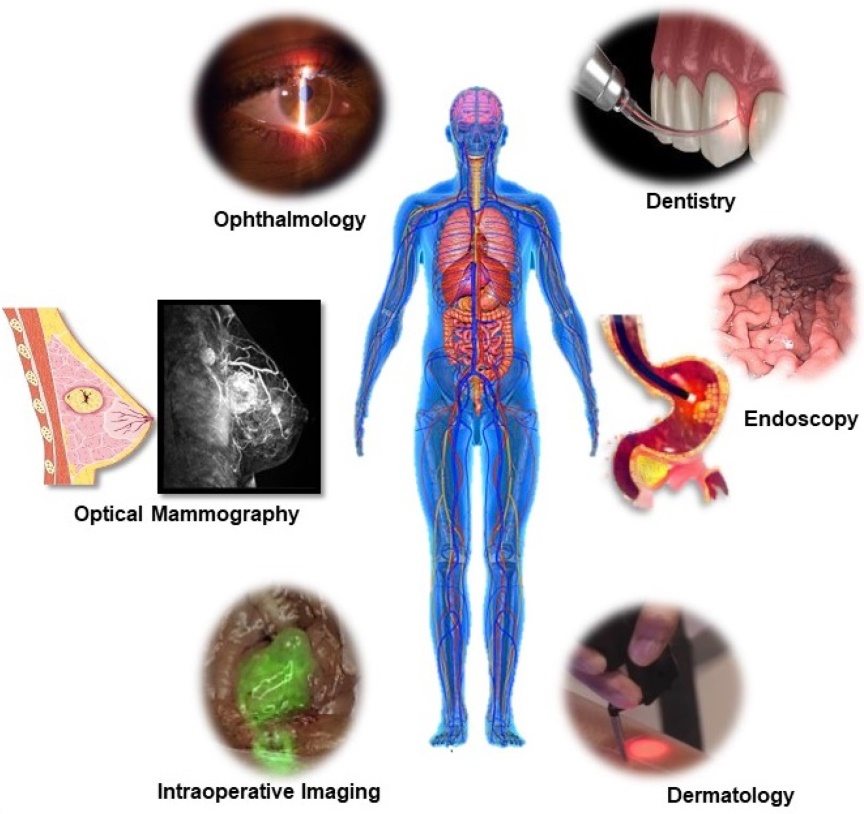
** Medical application of Laser Light.** Representative applications of light in the human body for diagnosis, imaging, surgery and therapy.

**Figure 5:**
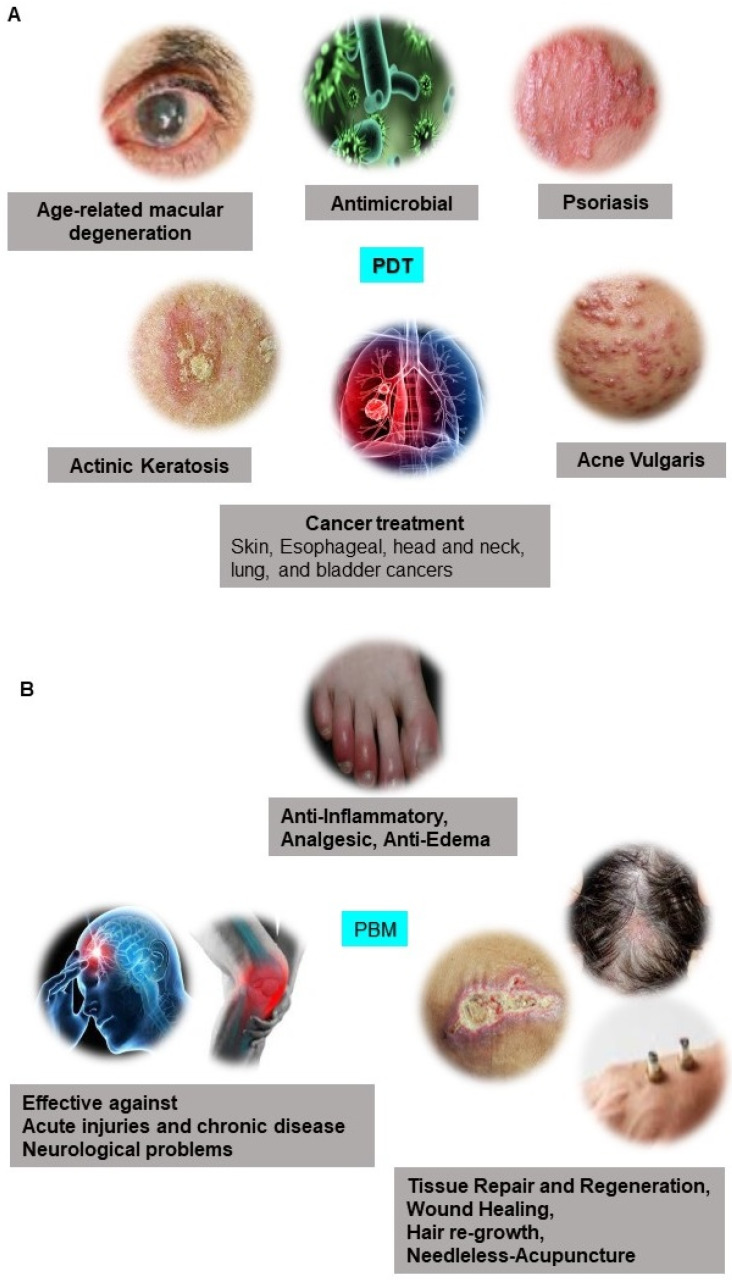
Medical application of Laser Light. Preclinical and clinical applications of A Photodynamic Therapy (PDT) and B Photobiomodulation Therapy (PBM).

The unique properties of laser light such as monochromaticity, coherence, directionality, high brightness, and generation of a focused narrow beam of light has made the applications of laser radiation in medicine so important. Being faster, less invasive, with better accuracy and a greater intervention effect, lasers have greatly revolutionized the field of medicine ranging from surgery and therapeutics to diagnostics which has been represented in Figure 4 and 5. Some of such medical field includes cardiology, dentistry, dermatology, gastroenterology, gynaecology, neurosurgery, orthopaedics, ophthalmology, otolaryngology, and urology. Additionally, laser also offers diagnostic applications such as laser-induced fluorescence (LIF) spectroscopy/imaging, Optical coherence tomography (OCT) and laser Doppler flowmetry (LDF). Lasers have also become an essential tool in other biological applications from high-resolution microscopy (confocal microscopy) to subcellular nanosurgery 42. Even after tremendous advances in different types and delivery systems of lasers in medical and biological field, in recent years there has been significant progress towards miniaturization their size to integrate into live cells as bio-derived/biological lasers as new forms of intracellular sensing, cytometry and imaging devices 45 In an attempt for miniaturization and cellular integration, main advancement has been made in the field of optical microcavities, structures that enable the confinement of light in microscale volumes, biomolecules or biocompatible molecules as gaining medium and reducing the size from the classical Fabry-Perot resonator, to novel classes of whispering gallery mode (WGM) microresonator 46,47.

### Biological lasers to intracellular living lasers and their biomedical applications

Biological lasers or biolasers make use of biocompatible, biological and/or natural molecule as lasing medium and/or part of its cavity for optical feedback and can also constitute passive optical components that tune the laser output by modulating the system's effective refractive index 48. These biolasers have the potential to generate coherent light sources compatible for living tissue integration. In recent years biological lasers have gained attention due to their potential to harness the amplifying power of stimulated emission from cell tracking to biosensing 49.

Although it sounds fascinating investigation of biological lasers started long back in 1970s. Arthur Schawlow's and Theodor Hänsch developed first ever edible laser by adding a dye, sodium fluorescein, to unflavoured gelatin 50. While in 1971, H. Kogelnik and C. V. Shank reported first distributed feedback (DFB) laser from rhodamine 6G doped gelatin 51. Besides, other such example of DFB biological lasers includes riboflavin (vitamin B2) doped gelatin 52 and fluorescent dye-doped silk 49,53,54. According to Yun et al., various fluorescent biomolecules are present in the human body and therefore can potentially be used as a laser gain medium. However, most of these biomolecules are excited by biologically harmful and less penetrating ultraviolet (UV) light and also have low quantum yields 47.

#### Importance and designing of biological lasers and cellular lasers

The need for investigating various life reactions within the living human body and single cell level has led to the development of molecular biosensing and bioimaging techniques. Such techniques depend on the reliable, sensitive, fast, and reproducible detection of the interactions between various biomolecules which allows understanding of the biological complexities, various disease processes, medical diagnosis, targeted therapeutics, and molecular biology 55,56. Fluorescent bioimaging is one of the very well-suited techniques for molecular bioimaging due to its activatable property, multiplex detection capabilities, high sensitivity, and low equipment cost. Many optical bio-probes such as fluorescent dyes and proteins, plasmonic nanoparticles and quantum dots have attracted the most attention and have been extensively explored for molecular bioimaging and biosensing 55-57. These fluorescent probes allow the non-invasive study of *in vivo* biological processes at the cellular and molecular level with the advantages of proving visible, quantitative and real time analysis. However, the broad emission spectra of these fluorescent probes limit their applications in high-resolution imaging, spectral multiplexing and ultra-sensitive bio-sensing.

**Figure 6:**
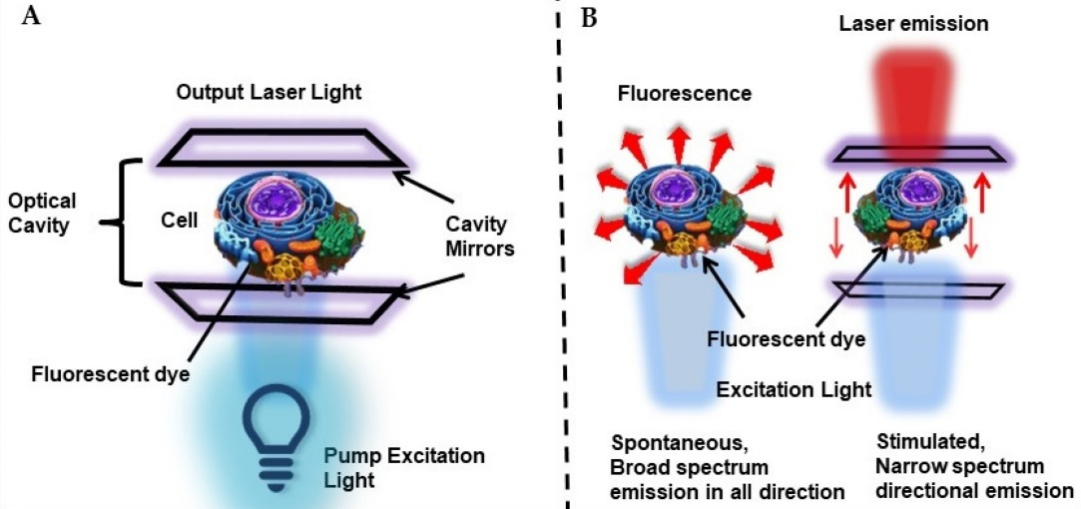
A Schematic representation of a cell laser. The system consists of a cell stained with fluorescent dyes or transfected with fluorescent proteins, placed between two high quality mirrors forming the optical cavity. B Comparison of fluorescence-based detection and laser-based detection.

To address this issue, intracellular laser probes, with the advantage of narrow bandwidth, strong coherence, and threshold-gated nonlinear emission were proposed 58. The concept of utilizing living cells as biological microcavity laser was initialized in 1990s, where for the first time Gourley and coworkers at Sandia National Laboratories utilized cells passively in a semiconductor laser, where the cell itself acted in light generating process as an internal component of a laser. They successfully demonstrated the concept of this laser technique with various living and fixed cells and probed human immune cells, between sickled and normal RBC shapes, distinguished between cancerous and normal cells 59-61. Later in 2011, Gather and Yun for the first time reported the demonstration of the first successful biological live cell lasers based on green fluorescent protein (GFP) and placing them between a pair of dielectric mirrors i.e. biological material as a viable gain medium for optical amplification 62. They used live eGFP-transfected 293ETN cells (human embryonic kidney cell line) and showed that single cell placed in a high- Q microcavity generates narrowband, directional and very bright laser emission, with characteristic longitudinal and transverse modes upon optical pumping with nanojoule/nano-second pulses. More importantly, even after a prolonged lasing process the lasing cells remained alive. Thus, Gather and Yun stated that many fluorescent proteins with excellent optical properties can act as promising gain media for stimulated emission and biolasing 62. Thus, principally biological laser is generated by introducing the biological material or system in a laser cavity, which acts as microresonators to induce narrow spectral stimulated emission (Figure 6A). This mode of *in vivo* optical amplification within biological systems have gained great importance for intracellular sensing, cytometry and imaging by overcoming the limitation of optical microscopy or fluorescence imaging modalities due to poor penetration depth of light in biological tissue and also due to better spectral, spatial, and temporal characteristics than fluorescence based imaging or detection as represented in Figure 6B 29,63,64. Thus, studies showed the significance of biological materials not only as laser gain media but also for forming the optical cavity for optical feedback and constituting the passive optical components to tune the laser output by modulating the system's effective refractive index.

**Figure 7:**
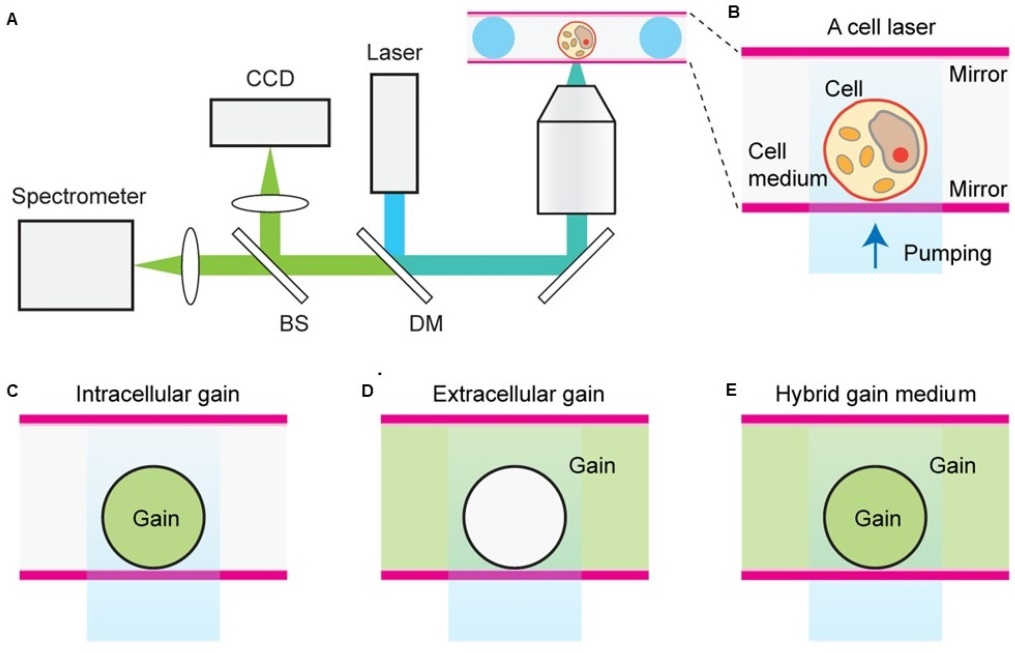
A and B Schematic representation of a cell laser with three different fluorescent dye gain configurations placed either C inside the cell, D outside the cell or E both inside and outside of the cell. Adapted with permission from 65, Copyright 2015 OPTICAL SOCIETY OF AMERICA.

Humar et al., demonstrated the comprehensive design principles of cell lasers using various fluorescent organic dyes as a convenient gain medium, cell types, and laser configurations (Figure 7). Importantly they showed that presence of dye gain medium inside or outside a cell, or in both, can be applicable to all cell types as well as offers flexibility in experimental design. Herein, number, cellular distribution and the emission and absorption cross-sections of the gain dye molecules determines the laser output characteristics.

Although fluorescent proteins had shown promising results as gaining materials but major issue with these proteins is their probability of eliciting immune response due to their foreignness. In this regard, Yun et al., demonstrated a miniature all-biomaterial laser, using U.S. Food and Drug Administration (FDA) approved biomaterials known as Generally-Recognized-AsSafe (GRAS) substances. They exploited the fluorescence property of intrinsic biomolecule Flavin mononucleotide (FMN) and vitamin B2 as a potential candidate for gain medium which showed high efficiency for permitting low lasing thresholds on the order of tens of nanojoules. Furthermore, vitamin encapsulated glycerol-mixed microspheres acted as a microdroplet-based whispering gallery mode (WGM) resonator 47. This work opened up the possibility of exploring various intrinsic fluorescent biomolecules and different device architectures for miniature biological laser applications.

Most of the studies in biological lasers mainly deal with different approaches and advancements in optics and optical devices to generate laser emission within the cell, but more important to this approach is how to couple the laser output with the intracellular or *in vivo* biophysical and biochemical processes. Depending on the applications, optical cavities can vary from planar to circular-shaped microsphere and ring resonators 30. Some of the optical microcavities used in biological laser and their applications have been represented in Table 3.

#### Intracellular lasers and applications

Besides designing and applications of biological lasers with the different biological lasing medium it is of great interest to translate *in vitro* biological lasers into *in situ* and *in vivo*, more like tissue microlasers. However, translational research suffers from several challenges which includes: (a) chemical stability in aqueous biological medium; (b) decreased refractive index contrast between the laser material and its surrounding; and (c) biocompatibility and biodegradability issues of potential lasing material, microresonator and optical pump. Further, the intracellular lasers should operate in the spectral region with minimum tissue absorption and scattering, and can easily be integrated with other *in vivo* bioimaging techniques and microscopy 100. For the first time, a “blood laser” was reported using a clinically acceptable concentration of indocyanine green (ICG) dye and a high *Q*-factor optofluidic ring resonator in human whole blood. ICG lasing was only accomplished when ICG binds to serological components such as albumin, globulins and lipoprotein 85. Humar et al. investigated the feasibility of implanting stand-alone laser particles into biological tissues to enable novel optical imaging, diagnosis and therapy. They employed approved and clinically used biocompatible materials i.e. transparent polymers poly(lactic-co-glycolic acid) (PLGA) and poly(lactic acid) (PLA) based solid microbeads of sizes 10 µm to 25 µm dopped with Nile red, which showed their suitability for operation both in soft solid tissues and in blood. Similarly, they demonstrated laser light emitting BODIPY based Cholesterol Bragg onion microdroplet lasers which also offer the possibility of *in vivo* temperature sensing 86.

**Figure 8:**
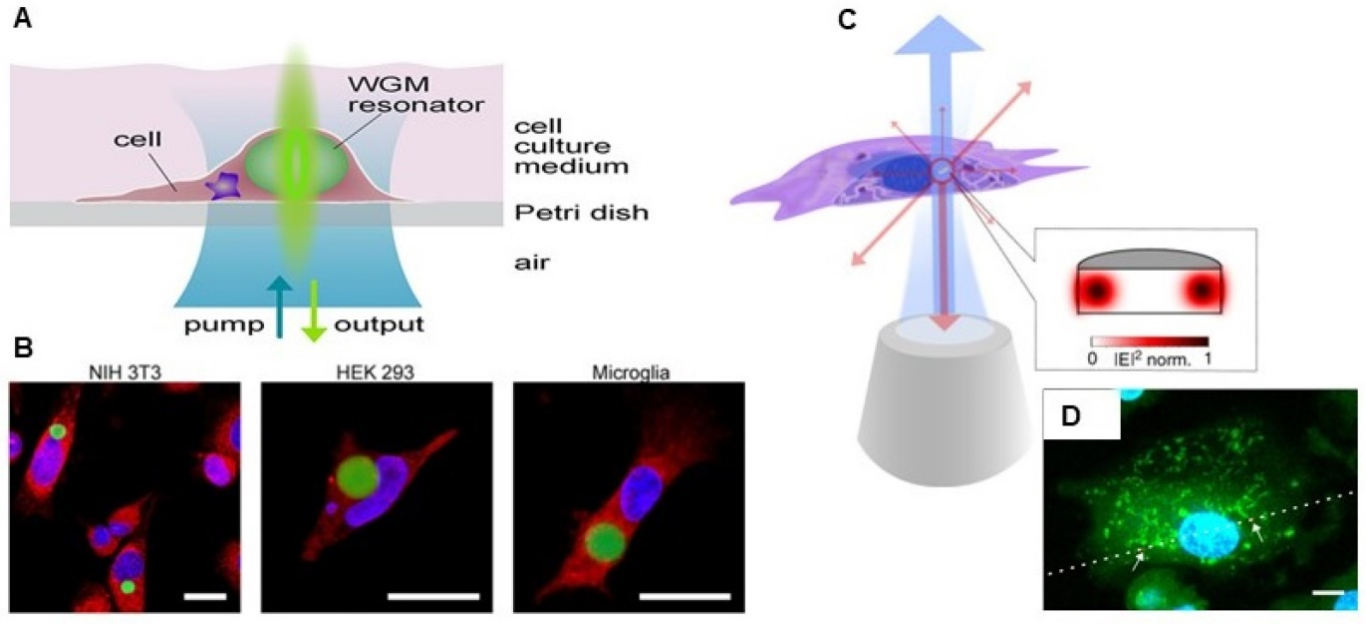
** A** Schematic illustration with self-sustained intracellular laser lasing from live cells containing intracellular optical resonators. **B** Confocal laser scanning microscopy image of NIH 3T3 fibroblasts, Human Embryonic Kidney 293, and primary mouse microglia internalized with polystyrene divinylbenzene (PS-DVB) microsphere resonators (green fluorescence). Adapted with permission from 101, Copyright 2015 AMERICAN CHEMICAL SOCIETY. **C** Schematic Illustration of a semiconductor nanodisk laser internalized into a cell, which is optically pumped through a microscope objective (blue) with laser emission (red) collected by the same objective.** D** Laser scanning confocal fluorescence microscopy image of fixed macrophage with internalized nanodisks (red fluorescence). Adapted with permission from 100, Copyright 2018 NATURE.

The real time cell tagging and tracking is one of the most reliable techniques for understanding the complexity of biological processes and systems down to single-cell analysis. Many studies have shown the utility of intracellular microlasers with spectral multiplexing capability and improved optical properties for cell tagging and tracking applications. Laser particles made up of silica-coated semiconductor microcavities, generating single narrowband emission peaks have been utilized for large-scale, comprehensive single-cell analysis. These stable and biocompatible Laser particles identified tagged cells at single cell level using Laser pArticle Stimulated Emission (LASE) microscopy in a 3D breast cancer cell tumour spheroid model 45. Intracellular delivery of WGM resonators in several different cell types, including mitotic, neuronal, macrophages, fibroblasts and primary cells have shown to facilitate barcode-type cell tagging and tracking of a large number of cells simultaneously. Moreover, as shown in Figure 8, these microresonators are reported to be stable, internalized and retained within the cell, even during the cell division process, thus enabling tagging of several generations of cells along with distinctively identifying and tracking individual migrating cells 100-102. Microlaser based cell tagging and tracking offer advantages over routinely used fluorescence-based approaches which limits the use of only a few fluorescent probes at a time due to their spectral overlap. In another study, fluorescent probe doped polystyrene WGM microspherical lasers were actively introduced into cardiac cells as a multifunctional optical biosensor to monitor cardiomyocyte contractility process at single cell resolution and long-term tracking of individual cardiac cell. High spectral sensitivity of WGM microlasers for the changes in its surrounding refractive index, was explored for monitoring the effect of nifedipine (Ca^+2^ channel blocker) on contractility of neonatal cardiomyocytes, real time measurement of complete contractility profiles for the beating heart in live zebrafish. Moreover, this have also been used as a robust contractility sensor at subcellular resolution, in thick living myocardial slices (~ 100s of µm) of rat heart tissue, which is even not achievable with multiphoton microscopes 103. Cadmium sulfide based nanowire biolasers internalized by macrophages were demonstrated to monitor the 3D migration of individual cells both *in vitro* and *in vivo* with enhanced contrast for dual-modality tracking and imaging via both OCT and fluorescence microscopy 104. For deep tissue imaging and labeling, NIR intracellular microlaser utilizing NIR gain materials have been developed. WGM microlasers made from (E)-3-(4-(diptolylamino)phenyl)-1-(1-hydroxynaphthalen-2-yl)prop-2-en-1-one (DPHP) coated silica shell microbeads exhibited NIR emission between 720 -790 nm and were used not only as a barcode-type cell tagging and tracking of millions of individual macrophages but also for monitoring the transformation of normal macrophages to foamy ones 105. Continuous-wave Tm^3+^-doped upconversion nanoparticle with energy-looping excitation mechanism coated onto polystyrene microbeads as WGM microresonators was developed by Fernandez-Bravo et al. These microlasers showed their lasing activity when submerged in serum with an emission at 980 nm when excited with 1064 nm light 106. Other than deep tissue penetration, NIR microlasers offer various other advantages such as low interference from auto-fluorescence along with minimal photo-damage to cells.

**Figure 9:**
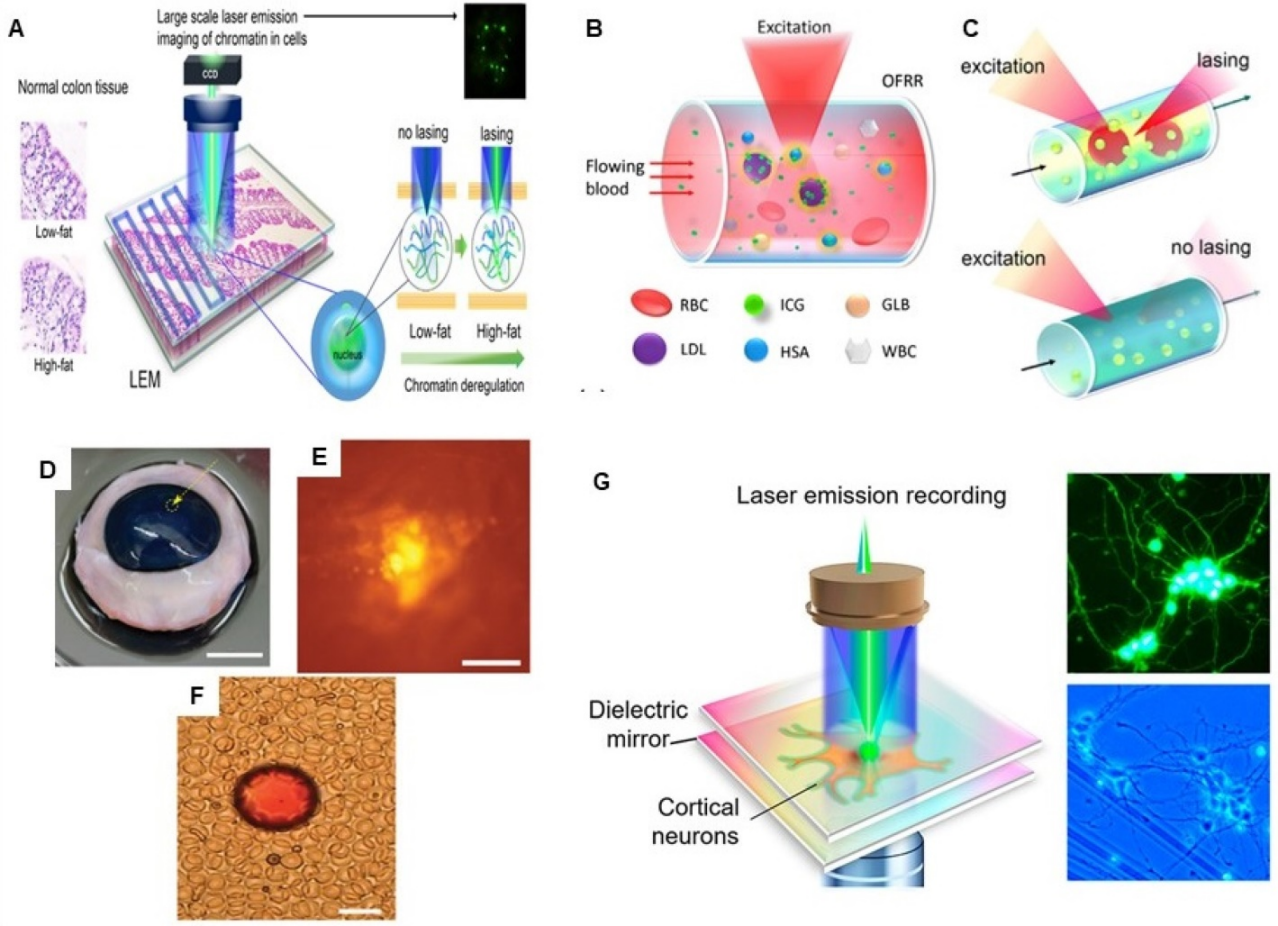
A Conceptual illustration of a large-scale rapid scanning laser-emission microscopy (LEM) for detecting pre-polyp nuclear abnormality by analyzing FFPE colonic tissues from mice with colon cancer risk. Here, a 10 µM thick FFPE tissue section is sandwiched within a high-Q Fabry-Pérot (FP) cavity. Adapted with permission from 75, Copyright 2019 OPTICAL SOCIETY OF AMERICA. B and C Schematic illustration of the Indocyanine green (ICG) laser using a high *Q*-factor optofluidic ring resonator (OFRR), in human serum and whole blood. ICG lasing can only be achieved when ICG binds to serological components such as albumin and lipoprotein. Adapted with permission from 85. Copyright 2016 OPTICAL SOCIETY OF AMERICA. Lasing of biodegradable polymer beads implanted in D bovine cornea, E skin and F blood. Adapted with permission from 86, Copyright 2017 OPTICAL SOCIETY OF AMERICA. G Conceptual illustration of the experimental configuration of a neuron laser are sandwiched inside an Fabry-Pérot (FP) cavity formed by two highly reflective mirrors to record the neuron activities with laser emission. Adapted with permission from 107, Copyright 2020 AMERICAN CHEMICAL SOCIETY.

Further, microlaser based lasing emissions have been used for imaging and mapping, where the light emitted by tiny intracellular laser particles can produce images to provide biomolecular and morphological information about the cells, particularly valuable for deep tissue imaging. For example, LASE microscopy offers the advantage of subdiffraction resolution, low out-of-focus background and 3D optical sectioning, required for imaging of thick biological tissues. LASE microscopy is fundamentally based on the principle of excitation of nano/micro- laser particles by a tightly focused optical pump beam with nonlinear power dependence stimulated emission of narrow spectrum. LASE microscopy offers the advantage of a standalone submicron size laser, where it uses single-photon absorption (although two-photon pumping is also possible) to generate stimulated emission only at the focal volume without the need of a pinhole or complex illumination or detection schemes as required in confocal laser scanning microscopy and two-photon microscopy 76. Chen and collaborators proposed the concept of scanning Laser-Emission-based Microscope (LEM), which is integrated with a 2D scanning stage to map a tissue labeled with site specific fluorophores and/or antibody-conjugated fluorophores sandwiched inside an FP microcavity formed by two mirrors and whereby, the images are constructed by scanning the pump beam across the whole tissue. LEM has been utilized for multiplexed lasing of various nuclear proteomic biomarkers (nucleic acids, EGFR, p53, and Bcl-2) in frozen Stage I/II human lung cancer tissues with a subcellular and sub-micrometer resolution, which is way far better than conventional fluorescence techniques. Moreover, LEM enables the distinction between cancer and normal tissues with high sensitivity and specificity for early stage cancer diagnosis 74. This LEM lasing system was further modified by microfabricating a SU8 spacer with a fixed height on the top mirror of the FP cavity to generate a robust platform for imaging in thick formalin-fixed, paraffin-embedded (FFPE) tissues. With the optimized lasing thresholds, this modified LEM system was able to differentiate cancer and normal FPPE biopsy tissues of colon, stomach, and breast cancer 73. This group also demonstrated the utility of LEM in detection of chromatin abnormality in colon tissue, despite the absence of observable lesions, polyps, or tumors in dietary controlled mouse model 75. All these studies showed the promising application of LEM for early tumor detection in clinical diagnosis even at the level of biomolecular changes linked with cancer development. Figure 9 represents some examples of tissue-based sensing with biological microlasers.

Other than cancer detection, Chen et al., employed LEM for *in vitro* monitoring of as low as nM concentration of Ca^2+^ ion dynamics and activities in single neurons and massive neuronal networks. Compared to a conventional fluorescence-based recording, LEM based recording showed about 100 times enhancement in sensitivity to detect intracellular Ca^2+^ ion fluctuations 107. As progress to the field of biological micro/nano laser-based imaging, monitoring and bio-inspired sensing, a novel concept of bioresponsive microlasers based on WGM nematic liquid crystal microdroplet have been proposed to exploit its interfacial energy transferability. This study demonstrated that interfacial energy transfer at the cavity biointerface and varying the nonradiative Förster resonance energy transfer (FRET) pair concentration ratios between the analytes (acceptor) and droplet (donor) can be utilized to understand how molecules can interact with and modulate the laser light. As a proof of concept, they demonstrated protein-based and enzymatic-based interactions occurring at the surface of microdroplets and whereby the fluorescence emission acts as interfacial laser gain medium and results into generation of different adjustable lasing wavelengths. This concept laid the foundation for the development of advanced tunable photonic devices at the molecular level by utilizing intracellular biological lasers 108.

## Optoelectronics in Medicine

Optoelectronics which is often considered as a subdiscipline of photonics, combines both photons and electronics and deals with applying electronic devices to the sourcing, detection and control of light. Optoelectronic devices are transducers that basically rely on light-matter interactions and electronic properties of matter to convert light into an electrical signal or vice versa. Optoelectronic devices are complex systems combining optics, electronics, mechanics and software all in the same system and involved in study of light emission and detection using light sensitive devices and light generating components 109. Recent progress in optoelectronics devices have led to developments in two types of optoelectronic devices; wearable and implantable. These optoelectronic devices provide better scope for light delivery into inaccessible targets and allow continuous monitoring of health. Most of the optoelectronic devices presently being used in medical field includes wearable, battery operated and/or hard-wired personal healthcare devices like pulse oximeter, blood glucose monitor, heart rate monitor, urine analysis, measuring the amount of oxygen in the blood, dental colour matching and exhaled biomarkers monitoring 110-112. Further, with advancements in safe and biocompatible biomaterials, health monitoring technologies are now being focused on wireless, battery free wearable optoelectronic devices with soft “skin-like” properties. Precommercial prototypes of these devices make use of near-field communication (NFC) technology for multicolor light emission and detection, which allows precise measurements of the optical properties of the skin and also provide information on temporal dynamics of blood flow, heart rate, tissue oxygenation to evaluate color of the skin, and/or of color-responsive materials for environmental detection 113. Other than the wearable devices, flexible optoelectronic devices are incorporated into multifunctional endoscopes, by integrating transparent bioelectronics with theranostic nanoparticles to combine imaging and therapy. These multifunctional endoscopes showed promising *in vivo* optical fluorescence-based mapping, contact/temperature monitoring, pH and electrical impedance sensing, radio frequency ablation and localized photo/chemotherapy 114. Thus, it's being speculated that the future optoelectronic wearable devices possibly will integrate light based therapeutic functionalities.

### Implantable optoelectronic devices

Implantable optoelectronic systems that can be easily integrated within the body provides a promising diagnostic and therapeutic potential for basic research and clinical medicine 32,115. Unlike traditional bulk wearable optoelectronic devices, implantable ones can be placed in any spatially-isolated, curved regions within human bodies. In the field of implantable optoelectronics, a great progress has been achieved with prosthetic retinas, a promising approach to overcome the loss of photoreceptors and restore vision in patients with retinal degenerative diseases such as Age Related Macular Degeneration and Retinitis Pigmentosa 116. However, to enable implantation of biomedical electronic systems in the human body, these devices need to be miniaturized to mm scale i.e., microimplants, which will further overcome the issues with conventional surgical implantation. Although advances in bio-optical interface and semiconductor technologies have allowed integration of oscillators, electrodes, memory and wireless communication systems, on tiny miniatured silicon chips, still, miniaturization of the power source, for storage, harvesting, or transfer of energy deep into the body remains as a major challenge. This limitation is overcome by a process termed midfield powering by which a high-energy density region can be created to transfer milliwatt levels of power to ~5 cm deep into the tissue, sufficient enough to remotely drive wireless tissue microimplants 117. Other novel innovative designs in advanced electronics like microscale inorganic light-emitting diodes (LEDs), photodetectors and organic optoelectronic materials, hold great promise for *in vivo* biomedical applications 118-120.

Over the past recent years, the importance of self-powered flexible inorganic optoelectronic systems as next-generation electronics has gained interest as biomedical implantable optoelectronics due to their biocompatible, lightweight, self-sustainable, and thin properties. These self-powered flexible inorganic devices include batteries, LEDs, sensors, high-density memories, energy harvesters, and large-scale integration. The most attractive property of such self-powered optoelectronics that have drawn great interest is the utilization of a flexible energy harvester that can generate electricity by harnessing kinetic and mechanical energy from human muscular movements and can charge a flexible battery. Thus, it is being speculated that these systems can permanently operate in heart, diaphragm, and shoulders using their sustainable muscular movement energy. This will avoid the need for invasive operated replacement of battery and/or use of an external energy supply in implantable electronic systems over time. Such prototypes hold great promise in the future implantable biosensors, self-powered cardiac pacemakers, phototherapeutic or optogenetic tools for wireless remotely controllable *in vivo* monitoring of vital biophysical, electrophysiological, biochemical signals and further can be extended for cardiac/brain disorders treatments 121,122. The advanced miniaturized optoelectronic components widens the scope of application of optical biopsy via light-based spectroscopy for more accurate *in situ*, real-time evaluation and differentiation between malignant tissues from benign tissues. In this respect, Lee et al., engineered and reported a proof-of-concept miniaturized prototype optoelectronic sensor compatible with a 19-gauge fine-needle aspiration needle for rapid, volumetric, and quantitative multisite tissue optical sensing for pancreatic tissue assessment. This prototype was proposed to a promising step towards overcoming the challenges of accurate pathological diagnosis of suspected pancreatic neoplasm 123.

### Cellular-scale free to roam optoelectronic devices for optogenetics

**Figure 10:**
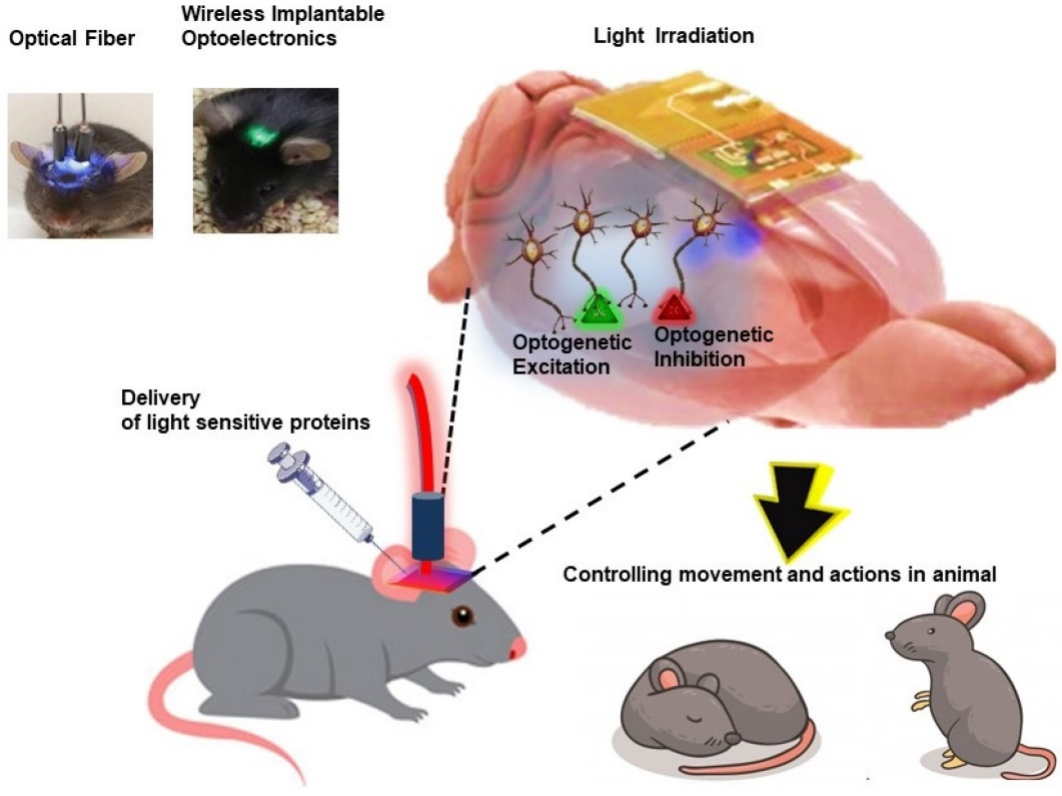
Schematic illustration of application of optoelectronics in optogenetics for understanding and modulating neural functions and animal behavior in a remotely controlled fashion.

Other than conventional light-based imaging, diagnosis and therapies, novel concepts in bio-optics and optoelectronic systems have revolutionized the field of neuroscience with the introduction of optogenetics approach. Broadly, the term “optogenetics” refers to an approach which involves precise control and monitoring of biological functions in cells, tissues, organs and organism by making use of genetic engineering and optical technology (Figure 10). This approach involves intracellular expression of genetically modified photosensitive proteins, which serves two functions: (1) optical sensor, allowing fluorescent readout to monitor changes in biological activities, and (2) optical actuators, which allow light to manipulate the cellular biological functions 124. Due to its non-invasiveness, high temporal and spatial resolution, optogenetics offers the advantage of precise study and manipulation of brain circuit functions to gain more insight on neurodegenerative disorders and injuries along with development of targeted therapy 125. Genetically modified light-activated transmembrane proteins, opsins are most widely used optical actuators to control neural activity and cellular signaling upon light exposure. Blue light-activated cation channel protein channelrhodopsins 2 (ChR2) and yellow light-activated anion-conducting protein halorhodopsin are two most commonly used opsins to excite and silence neurons, respectively 125. For optogenetics, it is critically important to precisely deliver light into tissues with desired intensities. The most common and simplest way of light delivery involves implantation of optical fibers in the brain which are physically tethered to external light sources. However, use of tethered, bulk sized light source limits the free movement of the animal, thus altering bodily and neuronal behavior along with other limitations of lack of cell-type specificity and targetability for modulating small numbers of neurons and damaging effects to surrounding healthy tissues 126. Thus, to overcome these constraints field of optogenetics is focusing on more advanced wireless, miniaturized fully implantable subdermal battery free optoelectronic devices.

As a foundational work, Kim et al., reported ultrathin multifunctional all-in-one optoelectronic system of single cell dimension which incorporated independent and inorganic μ-LEDs collocated with optical, thermal, and electrophysiological sensors and actuators. These systems were easily mounted on releasable injection needles for insertion in deep tissue of brain in mice model 32. Following this pioneering breakthrough in optoelectronics, many groups have reported development and application of wireless, battery-free, injectable micron scale optoelectronics in understanding and modulating neural functions and animal behavior in a remotely controlled fashion 127-129. Further, Montgomery et al., reported an easy-to-construct, fully internal smallest (20 mg, 10 mm^3^) implantable wireless optogenetic system with minimal tissue heating upon optogenetic stimulation. Using this system, they demonstrated wireless optogenetic stimulation of central nervous system (brain) as well as peripheral nervous system (spinal cord and peripheral nerve endings) in mice model 130. Further, Park et al., demonstrated miniaturized, soft wireless optoelectronic systems with fully implantable, stretchable property to modulate peripheral and spinal neuronal circuits in freely moving animals, in an effort to advance the application of optogenetic studies for issues related to chronic pain, itch and other neurological disorders other than brain 131.

## Conclusion and Perspectives

In recent years, biophotonics have witnessed tremendous transdisciplinary discoveries in the field of clinical diagnostics to therapeutics. Here we have discussed two advanced and promising areas of biophotonics: intracellular biological laser and cellular scale optoelectronics. Biological microlasers offer the advantages of better performance at deep tissue depth due to their high sensitivity, bright narrowband emission, single-cell specificity, high signal to noise ratio, low background autofluorescence and minimum scattering interferences. Thus, they have shown to be potential in imaging, sensing, *in vivo* cell tagging as well as for long term tracking to gather information from biological processes occurring at the molecular to subcellular and cellular level, as well as in small animals. Application of biological microlasers will certainly advance our understanding of important biological processes like cancer metastasis, neuronal network development, wound healing, and immune response. Future designs in the field of optoelectronic applications in optogenetics will allow bioelectric signal-based sensing and power control for simultaneous stimulation of multiple and deep neural targets outside brain, spinal cord and peripheral nerve. Thus, this approach holds great promise in the treatment of several neurodegenerative diseases.

Both fields are still in their basic research phase, they need to overcome several limitations mainly with the biocompatibility issues, before proceeding with clinical translations. Other than biocompatibility issues few more challenges in the field of biological microlasers need to be addressed such as surface passivation, heat management, lowering the exposure of pumping light to avoid damage to biomolecules/cells, advanced calibration, broadening the range of fluorescence detection limits. Although in the area of optoelectronics, advancement in electronics now allows their easy delivery into any site of body via syringe injection, still the power supply, size and heat generation of optoelectronic implants needs to be taken care off. In near future, it is being envisaged that integration of self-powered and machine learning technology in portable wearable optoelectronic systems will allow *in vivo* “recognition,” “think,” “analyze,” “decide” and “control” abilities to assist in smart diagnosis, drug transport followed by controlled drug delivery. Fast growing research and advancement in technology will surely increase the integration of these light-based functionalities in patients for early detection and real time diagnosis of several diseases and designing of personalized treatment with better therapeutic outcomes. It's exciting to see how the biophotonics field will evolve in the future as a smart human health monitoring and therapeutic strategy.

## Figures and Tables

**Table 1:** Commonly used Lasers for medical applications

Lasing Medium	Laser Type	Wavelength (nm)	Penetration depth in tissue
Gas	Helium Cadmium (HeCd)	325	8 μm
		442	0.3 mm
	Argon ion (Ar)	488	0.8 mm
		514.5	1 mm
	Krypton ion (Kr)	530.9	1.1 mm
		568.2	1.6 mm
		676.4	5 mm
	Helium-Neon (HeNe)	632.8	3.5 mm
	Carbon dioxide (CO_2_)	10600	20 µm
Liquid	Dye	400-500	0.1-0.9 mm
		550-700	1-5 mm
Solid state	Potassium titanyl phosphate (KTP)	532.0	1.1 mm
	Neodymium:yttrium aluminium garnet (Nd:YAG)	1064	4 mm
	Neodymium-doped yttrium aluminum garnet (Nd:YAP)	1080	4 mm
		1341	4 mm
	Holmium: yttrium aluminium garnet (Ho:YAG)	2100	1 mm
Chemical	Hydrogen Fluoride (HF)	2600-3000	300-1µm
Semiconductor	Gallium aluminum arsenide (GaAlAs)	780	7 mm
		820	8 mm
		870	7 mm

**Table 2:** Wavelengths of Laser used for medical applications

Medical Applications	Wavelengths (nm)
Dentistry	465, 810-980
Hyperthermia of tumors	940, 980, 1064
Photobiomodulation Therapy (PBM)	465, 630, 635, 652, 660-690
Photodynamic therapy (PDT)	630, 635, 652, 660-690, 730, 753
Surgery	800-1500

**Table 3:** Commonly used Optical Microcavities of Biolasers for biological applications

Optical Microcavities	Description	Applications
Fabry-Pérot (FP) cavity	Constructed using a pair of parallel plane mirrors, sandwiching the gain medium between them. This allows bulk interaction between light and the gain medium, enhancing the direct interaction of light with matter.	Optofluidic laser-based detection 66-69 Single Cell based intracellular sensing, cytometry and imaging 58,62,70,71,65 Tissue lasers 72,73 Scanning “laser-emission-based microscope” (LEM) for detection of specific intracellular biomarkers 74,75 Laser pArticle Stimulated Emission (LASE) microscopy 76
Whispering Gallery Mode (WGM) cavity	The WGM cavity relies on the total internal reflection at the curved boundary where the light rays are trapped inside the cavity circulating along the curved boundary. WGM microcavities have been constructed in various forms starting from circular shaped capillaries, microdisks, microdroplets, microspheres, solid cylinders, ring-shaped waveguides to rectangular shaped rods.	Biosensing, Cell tagging and tracking 45,77-82 Studying Protein-protein interactions and protein-drug interactions 83 Chlorophyll based optofluidic laser 84 Lasing in human blood 85 Implantable tissue lasers 86 Biomolecule detection system 87
Distributed Feedback Laser (DFB) cavity	DFB microcavity consist of periodical structures to provide optical feedback with the gain material.	Lasing 52 Membrane lasers 88 Biosensing 89
Random Laser (RL) cavity	A RL does not have a fixed optical cavity and the optical feedback is provided by multireflections from scattering particle dispersed in the lasing active material.	Tissue mapping 90-92 Label-free biofluidic lasers 93 Tissue Laser 94,95 Lasing 96,97 Biosensing 49,98 Opto-chemical therapies 99
